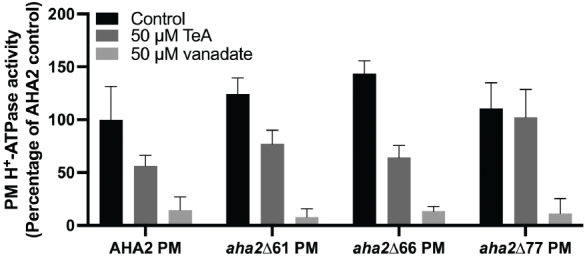# The mechanism behind tenuazonic acid-mediated inhibition of plant plasma membrane H^+^-ATPase and plant growth

**DOI:** 10.1016/j.jbc.2024.107167

**Published:** 2024-03-13

**Authors:** Nanna Weise Havshøi, John Nielsen, Anja Thoe Fuglsang

**Affiliations:** 1 Department of Plant and Environmental Sciences, Faculty of Science, University of Copenhagen, Frederiksberg, Denmark; 2 Department of Drug Design and Pharmacology, Faculty of Health, University of Copenhagen, Copenhagen, Denmark

**Keywords:** plasma membrane H^+^-ATPase, tenuazonic acid, fusicoccin, Arabidopsis thaliana, natural compounds, phytotoxin, AHA2

## Abstract

The increasing prevalence of herbicide-resistant weeds has led to a search for new herbicides that target plant growth processes differing from those targeted by current herbicides. In recent years, some studies have explored the use of natural compounds from microorganisms as potential new herbicides. We previously demonstrated that tenuazonic acid (TeA) from the phytopathogenic fungus *Stemphylium loti* inhibits the plant plasma membrane (PM) H^+^-ATPase, representing a new target for herbicides. In this study, we further investigated the mechanism by which TeA inhibits PM H^+^-ATPase and the effect of the toxin on plant growth using *Arabidopsis thaliana*. We also studied the biochemical effects of TeA on the PM H^+^-ATPases from spinach (*Spinacia oleracea*) and *A. thaliana* (AHA2) by examining PM H^+^-ATPase activity under different conditions and in different mutants. Treatment with 200 μM TeA-induced cell necrosis in larger plants and treatment with 10 μM TeA almost completely inhibited cell elongation and root growth in seedlings. We show that the isoleucine backbone of TeA is essential for inhibiting the ATPase activity of the PM H^+^-ATPase. Additionally, this inhibition depends on the C-terminal domain of AHA2, and TeA binding to PM H^+^-ATPase requires the Regulatory Region I of the C-terminal domain in AHA2. TeA likely has a higher binding affinity toward PM H^+^-ATPase than the phytotoxin fusicoccin. Finally, our findings show that TeA retains the H^+^-ATPase in an inhibited state, suggesting that it could act as a lead compound for creating new herbicides targeting the PM H^+^-ATPase.

Weeds contribute to 12% of crop losses annually in the United States alone (1). The herbicides used in agriculture today target a limited number of cellular processes in plants. This leads to an increase in genetic selection that favors herbicide-resistant weeds (2). Therefore, herbicides targeting different cellular processes may prove useful in agriculture. Specialized metabolites from fungi have been used for many decades in medicine, such as antibiotics. In recent years, the use of these metabolites as natural herbicides in agriculture has been investigated (3, 4). Researchers are searching for new herbicides that target other vital plant mechanisms to circumvent the increase in herbicide resistance (2, 4). When searching for new compounds with potential as growth-controlling agents, it is important to obtain a detailed understanding of the molecular mechanisms behind their interference with cellular processes. Such information is important for human safety, as their mechanisms must specifically target plants.

We previously screened for fungal compounds targeting the plant plasma membrane (PM) H^+^-ATPase (5). One such compound was tenuazonic acid (TeA) from *Stemphylium loti* extract. TeA is a non-host-specific natural compound found in *Stemphylium* spp., *Alternaria* spp., and several other plant-pathogenic fungi (5, 6, 7). TeA is biosynthesized from an isoleucine residue by a non-ribosomal peptide synthase and modified by a polyketide synthase (8). TeA was previously characterized as an inhibitor of photosystem II (9) but we determined that TeA inhibits the PM H^+^-ATPase at a lower concentration via a mechanism involving the C-terminal regulatory domain of this enzyme. However, the molecular mechanism of this inhibition has not been investigated in detail (5).

The inactivation or inhibition of plant PM H^+^-ATPase activity causes cell necrosis due to the disturbance of the electrochemical gradient across the PM. Several plant pathogenic fungi and bacteria target PM H^+^-ATPase to aid in their infection of plants. One mechanism is the use of small metabolites that directly and indirectly affect PM H^+^-ATPase activity. The most common mechanism is the indirect inhibition of the PM H^+^-ATPase activity by natural compounds that are inserted into the surrounding lipid bilayer and modulating its biophysical properties (reviewed in Havshoi and Fuglsang (10)). To date, TeA is the only natural compound from fungi that has been found to directly inhibit PM H^+^-ATPase activity.

The PM H^+^-ATPase is an important enzyme for cell growth, as it is responsible for acidification of the cell wall, which is known to contribute to cell expansion, as explained by the acid growth theory (11, 12, 13, 14, 15). PM H^+^-ATPase acidifies the cell wall by pumping H^+^-ions from the cytoplasm into the apoplast, against the pH gradient, via the hydrolysis of ATP. This results in an electrochemical gradient via the creation of an acidic environment in the apoplast. At the same time, this stabilizes the membrane potential across the PM. Secondary transporters in plant cells are facilitated by the membrane potential. Therefore, the PM H^+^-ATPase also an important player in the transport and uptake of macro- and micronutrients (16).

There are 11 isoforms of the PM H^+^-ATPase in *Arabidopsis thaliana* (17), which are known as AUTOINHIBITED PM H^+^-ATPase (AHA) isoforms. The most abundant isoforms are AHA1 and AHA2; AHA2 is mostly found in roots (18, 19). The regulatory mechanism of AHA2 has been intensely studied over the past 3 decades. The activation of AHA2 depends on the phosphorylation of the penultimate Thr947 and the binding of the regulatory 14-3-3 proteins (20, 21). AHA2 contains a long C-terminal regulatory domain of approximately 100 amino acid residues that take part the in autoinhibition of AHA2 enzyme activity (22). It is well established that two regions in the C-terminal domain are involved in binding to the central domain and a lesser extent to the N-terminus (23, 24). These two regions (Regulatory Regions I and II) were identified through an alanine scanning of all amino acid residues in the C-terminal domain to identify residues important for the regulation of AHA2 activity (25, 26). These studies revealed that mutating a single amino acid residue could lead to the formation of a hyperactivated AHA2, with increased phosphorylation of Thr947 and enhanced binding of 14-3-3 protein compared to the wild type (WT). Additionally, other phosphorylation sites of the C-terminal domain of AHA2 have been found to activate or inactivate the protein (27, 28, 29).

In this study, we investigated the inhibitory effects of TeA and a related compound on PM H^+^-ATPase activity and determined that TeA with an isoleucine backbone is the most potent isoform. TeA caused cell necrosis and significantly inhibited growth in *A. thaliana*, supporting the notion that this phytotoxin could be used as a natural herbicide. We showed that TeA inhibits PM H^+^-ATPase activity by binding to the C-terminal domain in Regulatory Region I, possibly stabilizing the binding between the C-terminal domain and the central domain and causing the pump to remain in the autoinhibitory state. Furthermore, we demonstrated that this inhibition is also affected by the phosphorylation status and binding of 14-3-3 proteins to the C-terminal domain, thereby altering the activity of the PM H^+^-ATPase.

## Results

### Tenuazonic acid causes cell necrosis and wilting in larger *A. thaliana* plants

**Figure 1:**
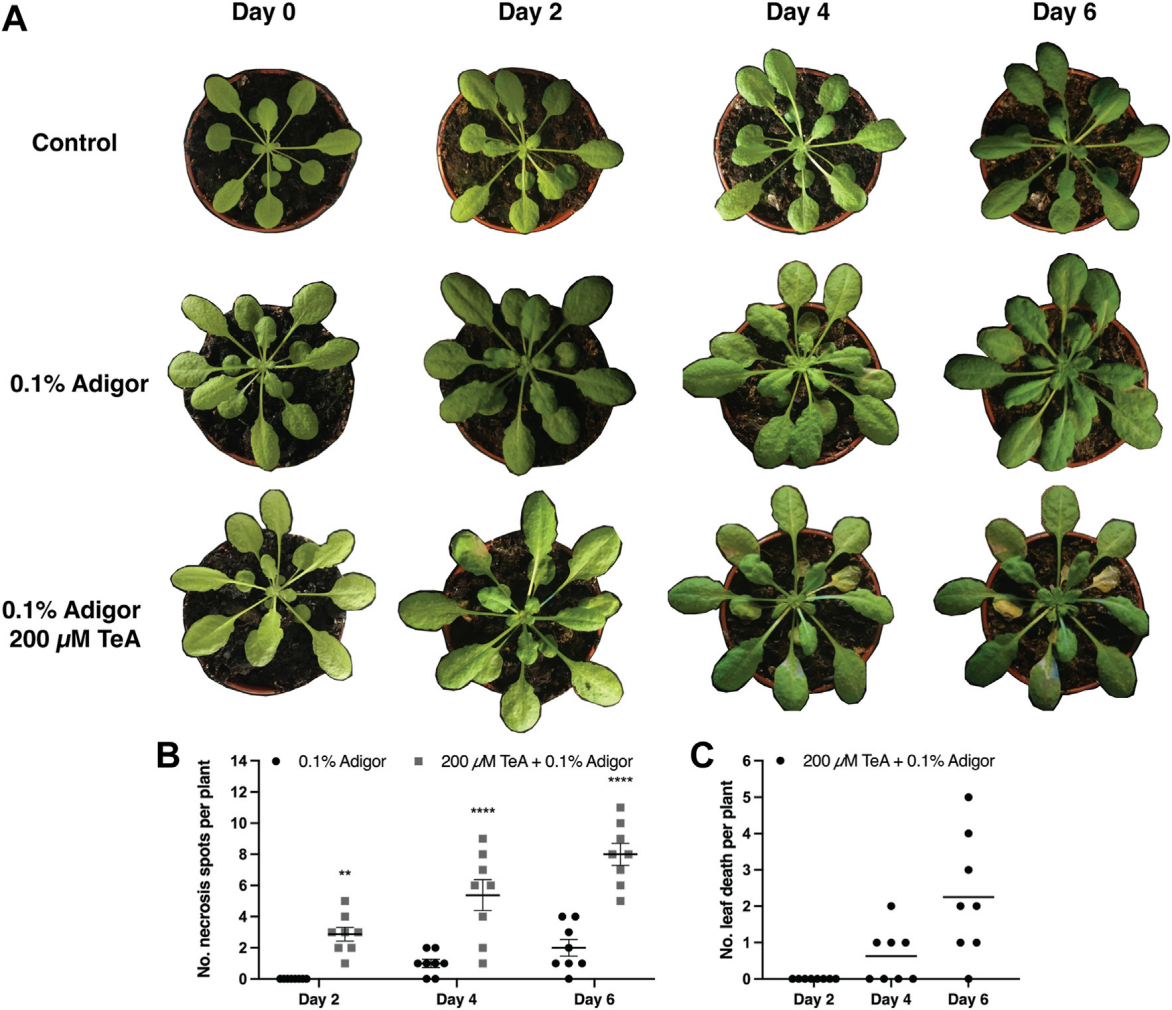
**Testing the potential use of tenuazonic acid (TeA) as an herbicide.***A*, *A. thaliana* plants were grown for 31 days and treated with 200 μM TeA + 0.1% Adigor, 0.1% Adigor, or water on days 0, 2, and 4. Adigor was used as an adjuvant to penetrate the leaf cuticle. Plants (*n* = 8) were treated on days 0, 2, and 4 and photographed right before treatment and on day 6. *B*, The number of necrotic spots on plants treated with 200 μM TeA + 0.1% Adigor or 0.1% Adigor on days 2, 4, and 6 post treatments. Data are presented as the mean number of necrotic spots per plant ±SEM. *C*, The amount of leaf death per plant treated with 200 μM TeA + 0.1% Adigor on days 2, 4, and 6 post treatments. Data are presented as the mean number of dead leaves per plant. Statistical data analysis was performed using two-way ANOVA with Bonferroni multiple comparison test in GraphPad Prism 9, *p* < 0.0332 (∗), *p* < 0.0021 (∗∗), *p* < 0.0002 (∗∗∗), *p* < 0.0001 (∗∗∗∗).

We investigated the potential use of TeA as a natural herbicide by treating 31-day-old *A. thaliana* plants with 200 μM TeA. Treatment was given on days 0, 2, and 4 to increase the potency of the phytotoxin. We used 0.1% Adigor as an adjuvant to penetrate the leaf cuticle, enabling TeA to enter leaf cells. We observed cell necrosis 2 days after the first TeA treatment (Fig. 1*A*), and all plants treated with TeA contained at least one spot with cell necrosis per plant (Fig. 1*B*). Cell necrosis increased over the course of 6 days, with whole leaves dying on the fourth day and most plants containing at least one dead leaf per plant 6 days after treatment (Fig. 1*C*). Treatment with Adigor alone led to the formation of some minor necrotic spots (Fig. 1, *A* and *B*) but did not cause any leaves to completely wither.

### Testing the potency of tenuazonic acid analogs on PM H^+^-ATPase activity

**Figure 2:**
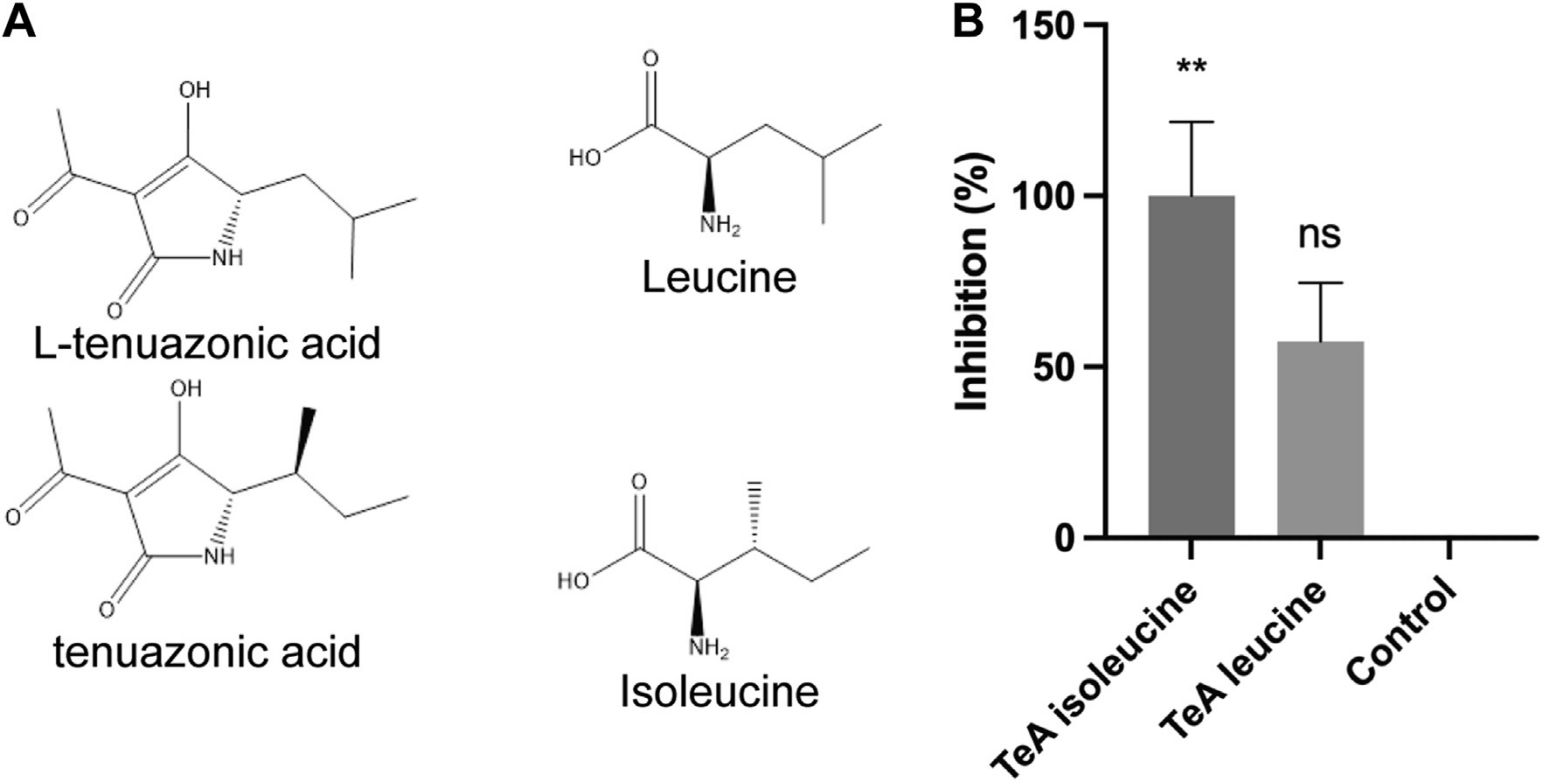
**Testing the potency of a tenuazonic acid (TeA) analog on inhibitions of plasma membrane (PM) H**^**+**^**-ATPase activity.***A*, chemical structure of TeA and L-TeA. *B*, *S. oleracea* PM vesicles were treated with 25 μM TeA or TeA analog to investigate their effects on ATPase activity at pH 6.5. Assays were performed in six technical replicates from three biological replicates (*n* = 18), and activity is presented as the mean percentage of inhibition ±SEM. Statistical data analysis was performed using one-way ANOVA with Bonferroni multiple comparison test in GraphPad Prism 9, *p* < 0.0332 (∗), *p* < 0.0021 (∗∗), *p* < 0.0002 (∗∗∗), *p* < 0.0001 (∗∗∗∗).

We tested the structural specificity of the interaction of TeA with the PM H^+^-ATPase by synthesizing another compound closely related to TeA. Since TeA is synthesized from an isoleucine backbone in fungi (8), we synthesized TeA as well as (*S*)-3-acetyl-4-hydroxy-5-isobutyl-1,5-dihydro-2*H*-pyrrol-2-one (L-TeA): TeA based on an isoleucine backbone, and L-TeA is based on leucine. We therefore obtained two nearly identical molecules with the same molecular weight but with different spatial properties due to different alkyl sidechains (Fig. 2*A*). We treated PM vesicles from *S. oleracea* with 25 μM of each of the two related compounds and measured their effects on ATPase activity. The synthesized TeA was a more potent inhibitor of ATPase activity than L-TeA (Fig. 2*B*). Treatment with TeA (synthesized) significantly reduced ATPase activity, whereas L-TeA had no effect on this activity. These results indicate that the position of the methyl group of the alkyl sidechain in the TeA molecule is required to bind and inhibit H^+^-ATPase activity.

### Cell elongation in root tips is inhibited by tenuazonic acid treatment

The phytotoxin fusicoccin (FC) constitutively activates the plant PM H^+^-ATPase, by stabilizing the binding between its C-terminal domain and 14-3-3 proteins (21, 30, 31, 32). We therefore investigated the potential competition between an activating compound (FC) and an inhibitory compound (TeA) of plant PM H^+^-ATPase. We grew *A*. *thaliana* seedlings for 6 days on a medium containing either 10 μM TeA, 10 μM FC, or a combination of both compounds and recorded the progression of roots and cell elongation over time.

**Figure 3:**
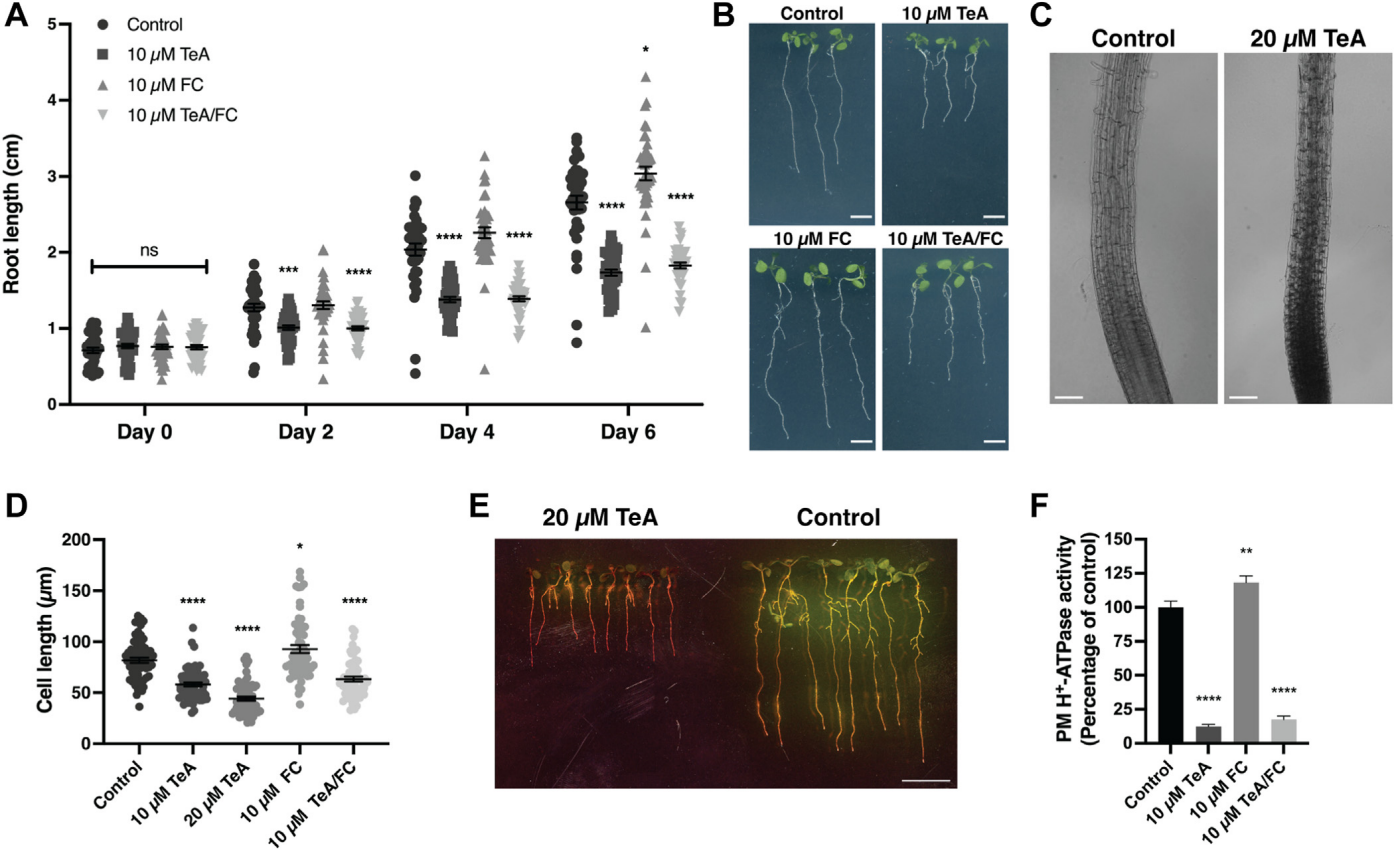
**Tenuazonic acid (TeA) prevents fusicoccin (FC)-induced activation of plasma membrane (PM) H**^**+**^**-ATPase.***A*, *A. thaliana* seedlings were grown for 6 days on ½× MS agar then transferred to fresh ½× MS agar containing 10 μM TeA, 10 μM FC, 10 μM TeA/FC, or control treatment, and incubated for 6 days. The root length of seedlings was measured every other day using Fiji ImageJ. Growth assays were performed in technical replicates of 20 from two biological replicates (*n* = 40), and root length is given in cm presented as mean ±SEM. *B*, *A. thaliana* seedlings on day 6 of treatment with 10 μM TeA, 10 μM FC, 10 μM TeA/FC, or control. Scale bar = 0.5 cm. *C*, root elongation zones of *A. thaliana* seedlings on day 6 of treatment with 20 μM TeA or control. Scale bar = 100 μm. *D*, length of cells in the elongation zone of seedlings treated with 20 μM TeA, 10 μM TeA, 10 μM FC, 10 μM TeA/FC, or control for 6 days. Cell length was measured using Fiji ImageJ, and growth assays were performed in replicates of 29 to 30 from two biological replicates (*n* = 59–60); root length is given in μm presented mean ±SEM. *E*, *A. thaliana* seedlings were grown for 6 days on ½× MS agar and transferred to fresh ½× MS plates with 20 μM TeA or control, incubated for 3 days, then transferred to Bromocresol Purple agar, and grown overnight before assessing acidification. The experiment was repeated three independent times with similar results. Scale bar = 1 cm. *F*, TeA/FC competition assay measuring the ATPase activity of *S. oleracea* PM H^+^-ATPase. Purified PM vesicles were pre-incubated with 15 μM TeA, FC, or TeA/FC at pH 6.5 for 10 min prior to assay start and assays were performed using 10 μM of phytotoxins. Assays were performed in technical replicates of three from two biological replicates (*n* = 6), and activity is presented as the mean percentage of non-treated samples ±SEM. Statistical data analysis was performed using one-way or two-way ANOVA with Bonferroni multiple comparison test in GraphPad Prism 9, *p* < 0.0332 (∗), *p* < 0.0021 (∗∗), *p* < 0.0002 (∗∗∗), *p* < 0.0001 (∗∗∗∗).

Root length was significantly reduced in response to TeA or combined TeA and FC treatment 2 days after transfer to a medium containing these compounds (Fig. 3*A*). Medium including only FC slightly stimulated root growth compared to seedlings from the control medium (Fig. 3*A*). Analysis of the cotyledons of seedlings showed that FC stimulated leaf growth, as cotyledons from treated seedlings were larger than those of the control (Fig. 3*B*). Seedlings with combined TeA and FC treatment also showed larger cotyledons than to seedlings treated with only TeA, even though root lengths under the respective treatments were approximately the same. Cell expansion in the root elongation zone was significantly reduced in seedlings treated with 10 or 20 μM TeA for 6 days (Fig. 3, *C* and *D*). FC treatment slightly enhanced cell expansion in the root elongation zone, but combined TeA and FC treatment significantly inhibited cell expansion to the same degree as treatment with TeA alone (Fig. 3*D*). Cells in the root elongation zone of seedlings treated with 20 μM TeA exhibited retarded growth, and cells accumulated in the elongation zone as new were formed from the meristem (Fig. 3*C*). We also investigated the effect of TeA on medium acidification using Bromocresol purple medium. Bromocresol purple is color-sensitive to changes in pH, changing from purple to yellow upon acidification. Seedlings treated with 20 μM TeA generated less acidification of the medium than the untreated control (Fig. 3*E*). Medium surrounding control seedlings turned bright yellow, whereas the surrounding TeA-treated seedlings remained slightly purple. These results suggest that the extrusion of protons from the root is inhibited by TeA.

### Activation of the plant plasma membrane H^+^-ATPase by the phytotoxin fusicoccin is blocked by tenuazonic acid

A previous study by Bjork *et al*. (5) revealed that TeA treatment could overcome pre-activation of the plant PM H^+^-ATPase by FC and that FC could not re-activate TeA-induced inhibition. This led us to further investigate the dynamics between TeA and FC. We used *S*. *oleracea* PM vesicles treated with 10 μM TeA, 10 μM FC, or a combination of both in an ATPase activity assay. ATP hydrolysis significantly increased (by 18%) in PM vesicles treated with 10 μM FC and decreased by 88% in vesicles treated with 10 μM TeA (Fig. 3*F*). Interestingly, the ATPase activity of the plant PM H^+^-ATPase decreased by 82% when treated with both TeA and FC at the same time. These findings suggest that the binding of TeA prevents the formation of an FC/14-3-3 complex and assures the inhibition of PM H^+^-ATPase activity.

### Tenuazonic acid requires the Regulatory Region I in the C-terminal domain of AHA2 in order to affect AHA2 activity

**Figure 4:**
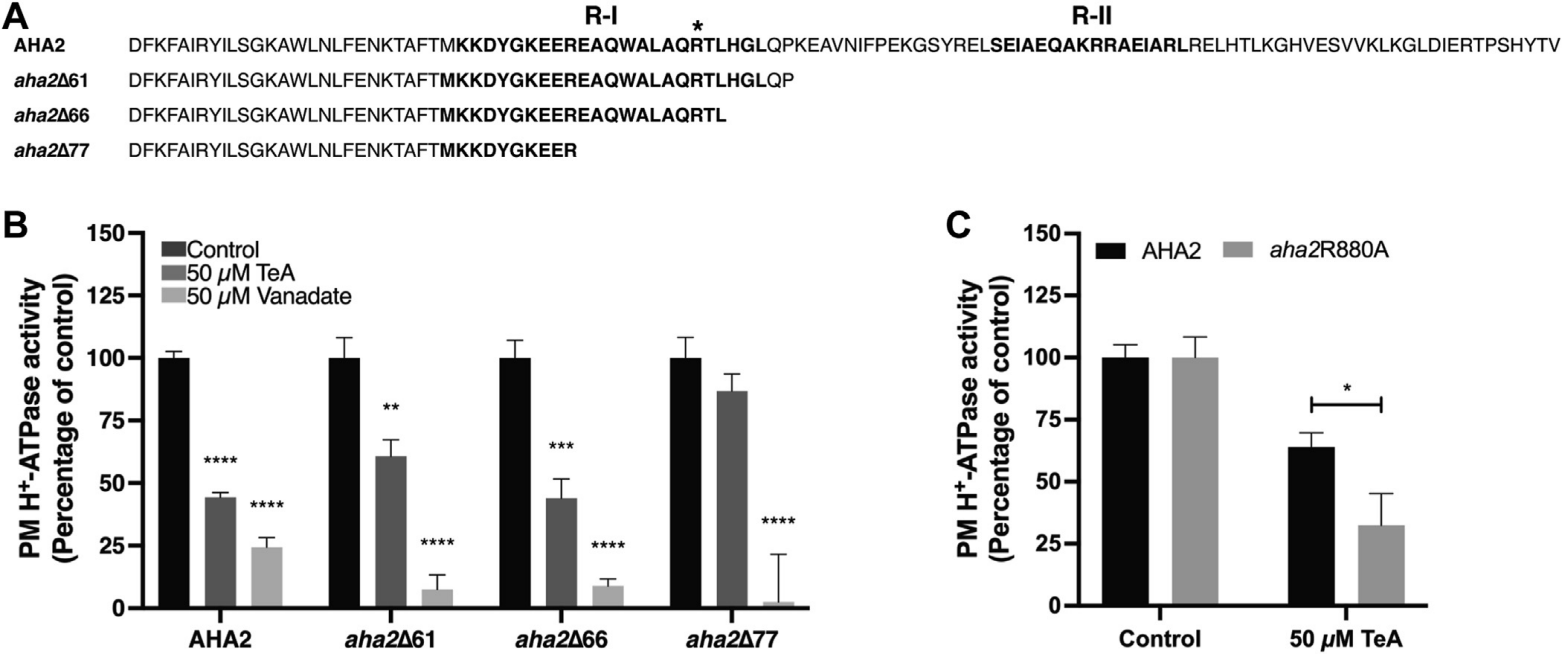
**Tenuazonic acid (TeA)-induced inhibition is dependent on the C-terminal domain of the plasma membrane (PM) H**^**+**^**-ATPase.***A*, amino acid sequence of the AHA2 C-terminal domain and truncated mutants *aha2*Δ77, *aha2*Δ66, and *aha2*Δ61. Amino acid residue R880 is marked with an asterisk (∗). Regulatory Region I (R-I) and II (R-II) are marked in bold. *B*, TeA-induced inhibition of the ATPase activity of AHA2, *aha2*Δ61, *aha2*Δ66, and *aha2*Δ77 purified from the PM fraction from *S. cerevisiae*. Membrane fractions were treated with 50 μM TeA or 50 μM vanadate (P-type ATPase inhibitor) at pH 6.5 for AHA2 and pH 7 for *aha2*Δ77, *aha2*Δ66, and *aha2*Δ61. The assays were performed in technical replicates of three from two biological replicates (*n* = 6), and activity is presented as the mean percentage compared to non-treated samples ±SEM. *C*, TeA-induced inhibition of the ATPase activity of AHA2 and *aha2*R880A purified from the PM fraction from *S. cerevisiae*. PM fractions were treated with 50 μM TeA in pH 6.5 for AHA2 and pH 7 for *aha2*R880A. Assays were performed in technical replicates of three from two biological replicates (n = 6) and activity is presented as mean percentage compared to non-treated samples ±SEM. Statistical data analysis was performed using Two-way ANOVA with Bonferroni multiple comparison test in GraphPad Prism 9, *p* < 0.0332 (∗), *p* < 0.0021 (∗∗), *p* < 0.0002 (∗∗∗), *p* < 0.0001 (∗∗∗∗).

**Supporting Figures 1:**
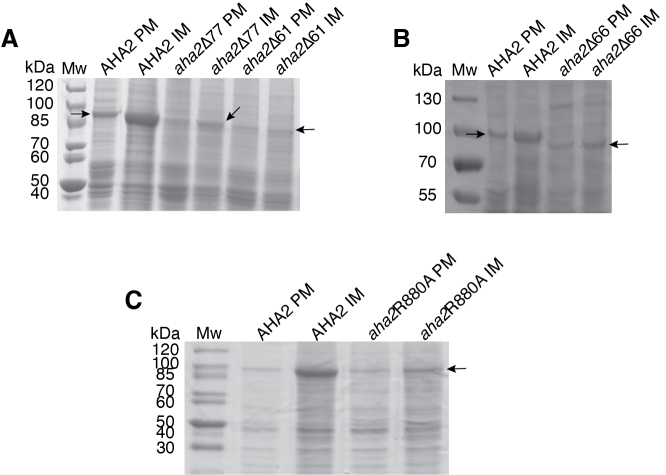


We employed *A. thaliana* AHA2 as a model enzyme to further investigate the inhibitory mechanism of TeA. We heterologously expressed AHA2 and its mutant forms in yeast (*Saccharomyces cerevisiae*) and purified the different membrane compartments. The binding of TeA was previously shown to be dependent on the C-terminal regulatory domain of AHA2 (5), which we investigate further. We expressed mutant proteins lacking part of the C-terminal domain (*aha2*Δ77, *aha2*Δ66, *aha2*Δ61) or harboring a C-terminal point mutation (*aha2*R880A) in *S. cerevisiae* (Fig 4*A*), and membranes were purified (Fig. S1) (22, 26). We then tested the effects 50 μM vanadate, a P-type ATPase inhibitor, has on the ATPase activity of these proteins, serving as a control inhibitor. Full-length AHA2 was significantly inhibited by TeA treatment, with a 55% decrease in activity (Fig. 4*B*), as were the two truncated mutants *aha2*Δ66 and *aha2*Δ61. By contrast, TeA had no effect on the activity of *aha2*Δ77 (Figs. 4*B* and S2). As expected, the activities of all versions of AHA2 were significantly inhibited by vanadate, with little or no activity observed (Fig. 4*B*). These results strongly suggest that one or more amino acid residues between *aha2*Δ66 and *aha2*Δ77 are required for TeA-induced inhibition of PM H^+^-ATPase activity. Residue Arg880, placed between *aha2*Δ66 and *aha2*Δ77, was previously shown to be important for the regulatory activity of the pump (26). The inhibitory effect of TeA on the ATPase activity of *aha2*R880A was more pronounced than on the activity of AHA2 (Fig. 4*C*). These results suggest that TeA binds to the plant PM H^+^-ATPase in the Regulatory Region I of the C-terminal domain and that changing Arg880 to an Ala residue increases TeA-induced inhibition of AHA2 activity.

### Tenuazonic acid-induced inhibition may require the plasma membrane H^+^-ATPase in its low-activity state

**Figure 5:**
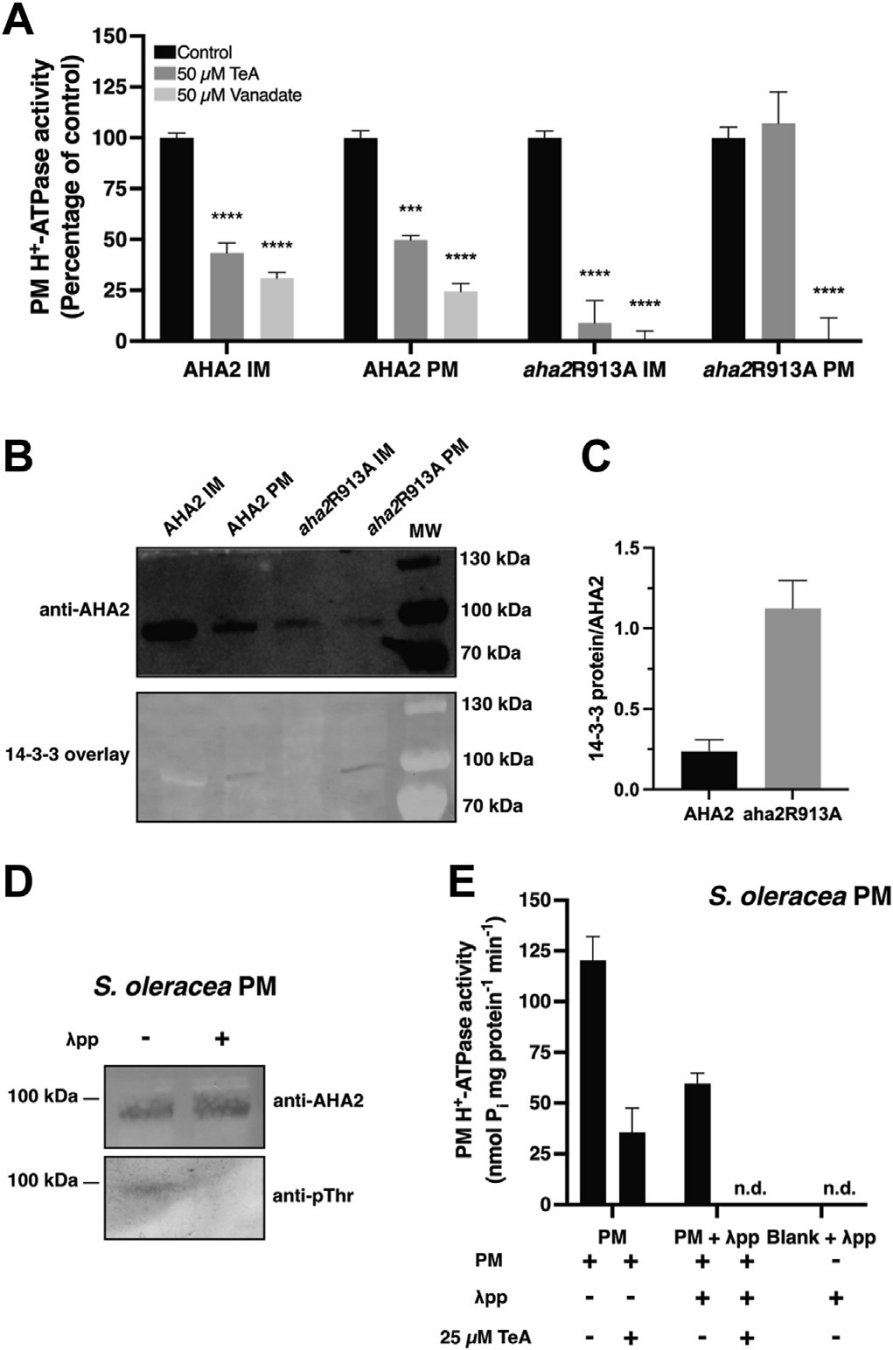
**Tenuazonic acid (TeA)-induced inhibition depends on the activity state of the plasma membrane (PM) H**^**+**^**-ATPase.***A*, TeA-induced inhibition of the ATPase activity of AHA2 and *aha2*R913A purified from the PM and internal membrane (IM) fractions from *S. cerevisiae*. Membrane fractions were treated with 50 μM TeA or 50 μM vanadate (P-type ATPase inhibitor) at pH 6.5 for AHA2 and pH 7 for *aha2*R913A. Assays were performed in technical replicates of three from two biological replicates (*n* = 6), and activity is presented as the mean percentage compared to non-treated samples ±SEM. *B*, immunoblot and 14-3-3 overlay blot of the AHA2 and *aha2*R913A PM and IM fractions to detect the binding ability of 14-3-3 proteins to these proteins. *C*, relative binding of 14-3-3 protein towards AHA2 and *aha2*R913A purified from the PM fraction. Overlay assays were performed in technical replicates of three (*n* = 3), and values are presented as relative ratios of the lane intensity from overlay blots and multiplied by protein levels from the immunoblot ±SEM. *D*, detection of PM H^+^-ATPase and pThr residues in the PM H^+^-ATPase by immunoblotting using PM vesicles from *S. oleracea* treated with and without λ-phosphatase (λpp). *E*, TeA inhibition assay of *S. oleracea* PM H^+^-ATPase treated with and without λ-phosphatase (λpp). PM vesicles were treated with 25 μM TeA at pH 6.5, and all assays were performed with technical replicates of six (*n* = 6). Non-detectable (n.d.) activity is indicated. Statistical data analysis was performed using two-way ANOVA with Bonferroni multiple comparison test in GraphPad Prism 9, *p* < 0.0332 (∗), *p* < 0.0021 (∗∗), *p* < 0.0002 (∗∗∗), *p* < 0.0001 (∗∗∗∗).

The characteristics of AHA2 and its variants have been well studied over the past few decades (22, 25, 26, 33). It is known that AHA2 expressed in *S. cerevisiae* will be present in both the PM fraction and in large quantities in the endoplasmic reticulum (ER), but the protein is only post-translationally modified when it is located in the PM (Fig. 5*B*) (33). Some amino acid mutations of the C-terminal domain of AHA2 give rise to increased phosphorylation of Thr947 and enhanced binding of 14-3-3 proteins: one such mutation is present in *aha2*R913A (25, 26).

We expressed and purified AHA2 and *aha2*R913A from the PM fraction and the internal membrane (IM) fraction of *S. cerevisiae* (Fig. 5*B*) to test the effects of TeA on AHA2 and the high-activity mutant *aha2*R913A. Analysis of the TeA-induced inhibition of AHA2 in the PM fraction and IM fractions (Fig. 5*A*) revealed 50% and 56% inhibition of ATP hydrolysis, respectively, with significant differences from the non-treated control. We detected 91% Inhibition of *aha2*R913A in the IM fraction in response to TeA treatment and complete inhibition in response to vanadate treatment. Interestingly, no TeA-induced inhibition of *aha2*R913A was detected when tested on the PM fraction. Furthermore, no ATPase activity was detected when this fraction was tested in response to vanadate. The *aha2*R913A mutant is a constitutively activated form of AHA2, since *aha2*R913A is highly phosphorylated at the Thr947 residue and is, therefore, able to bind more 14-3-3 protein than AHA2 (Fig. 5, *B* and *C*). In addition, both AHA2 and *aha2*R913A were unable to bind 14-3-3 protein when purified in the IM fraction but bound to 14-3-3 protein in the PM fraction (Fig. 5*B*), and *aha2*R913A bound more 14-3-3 protein than AHA2 (Fig. 5*C*) (26). These findings suggest that 14-3-3 protein binding and the activation of AHA2 are key components in TeA-induced inhibition of the plant PM H^+^-ATPase.

To further investigate this mechanism, we dephosphorylated PM vesicles isolated from *S. oleracea* using a specific Thr/Tyr/Ser phosphatase (λ-phosphatase) (Fig. 5*D*). The dephosphorylated PM vesicles showed a 2-fold decrease in activity compared to the control. Therefore, removing the phosphorylation from the PM H^+^-ATPase in *S. oleracea* reduced the ATPase activity of this enzyme, due to reduced binding of 14-3-3 protein. Inhibiting the dephosphorylated PM H^+^-ATPase with TeA led to no detectable ATPase activity (n.d.), whereas the phosphorylated control PM H^+^-ATPase was inhibited by 60% (Fig. 5*E*). λ-phosphatase showed no ATP hydrolytic activity by itself (Fig. 5*E*). These findings correspond with the activity of the heterologously expressed plant PM H^+^-ATPase, AHA2, suggesting that TeA-induced inhibition of the plant PM H^+^-ATPase is dependent on the activity state of the pump and on whether the C-terminal domain is phosphorylated and occupied by bound 14-3-3 protein.

## Discussion

### Tenuazonic acid causes cell necrosis in Arabidopsis

We tested TeA on larger *A. thaliana* plants to determine whether TeA could be used as a biological herbicide and as a proof-of-concept of the application potential of TeA. TeA caused cell necrosis within 2 days of treatment at low concentrations when combined with an adjuvant (Fig. 1, *A* and *B*) and caused several leaves to wither over the course of 6 days of continuous TeA treatment (Fig. 1*C*). An increase in TeA concentration may cause the overall death of larger *A. thaliana* plants. We also tested whether TeA at lower concentrations could penetrate the surfaces of plant leaves if combined with an adjuvant. The use of TeA as an herbicide has been extensively studied by Chen and Qiang (34). Their results point towards the use of higher concentrations of TeA or the development of an improved compound. A screening for TeA-based candidates that more efficiently inhibited the D1 protein of the photosystem II was recently performed by Wang *et al*. (35).

### The isoleucine backbone of tenuazonic acid is essential for its role in inhibiting the plasma membrane H^+^-ATPase

The use of TeA as an herbicide has recently been examined (34, 35). Wang *et al*. (35) tested the binding affinity of TeA and derivatives to the Q_B_ site of D1 protein from *A. thaliana* by molecular docking. Changing the position of the branched methyl group or adding more branched methyl groups to the side alkyl chain decreased the binding affinity of TeA to the Q_B_ site (35). We tested two compounds, one based on a leucine molecule (L-TeA) and one based on the isoleucine molecule, for their effects on PM H^+^-ATPase activity (Fig. 2*B*). In L-TeA, the branched methyl side group was moved to the next C-atom position of the molecule compared to TeA synthesized from isoleucine (Fig. 2*A*). However, a simple test showed that L-TeA had very low inhibitory activity on the PM H^+^-ATPase compared to TeA, indicating that the binding groove is very tight, allowing only limited room for changes. This corresponds to the results of molecular docking studies by Wang *et al*. (35), who showed that moving the branched methyl group changes the binding affinity of TeA toward both the PM H^+^-ATPase and the Q_b_ site of D1 protein.

### Tenuazonic acid retains PM H^+^-ATPase in an inhibited state, thereby decreasing cell growth

We previously investigated the effects of TeA and FC on the ATPase activity of PM H^+^-ATPase isolated from *S. oleracea* by using a biochemical competition assay (5). Here, to determine whether TeA can overcome FC-induced activation of this enzyme *in vivo*, we treated 6-day-old seedlings with TeA, FC, and a combination of both (Fig. 3*A*). TeA treatment significantly reduced root growth as well as leaf expansion (Fig. 3*B*) and reduced cell expansion in the root elongation zone (Fig. 3, *C* and *D*). In addition, 10 μM TeA treatment of PM vesicles inhibited the activity of *S. oleracea* PM H^+^-ATPase (Fig. 3*F*). Finally, medium acidification was reduced when *A. thaliana* seedlings were treated with 20 μM TeA (Fig. 3*E*). These results support the notion that TeA treatment inhibits cell growth by inhibiting PM H^+^-ATPase activity.

**Figure 6:**
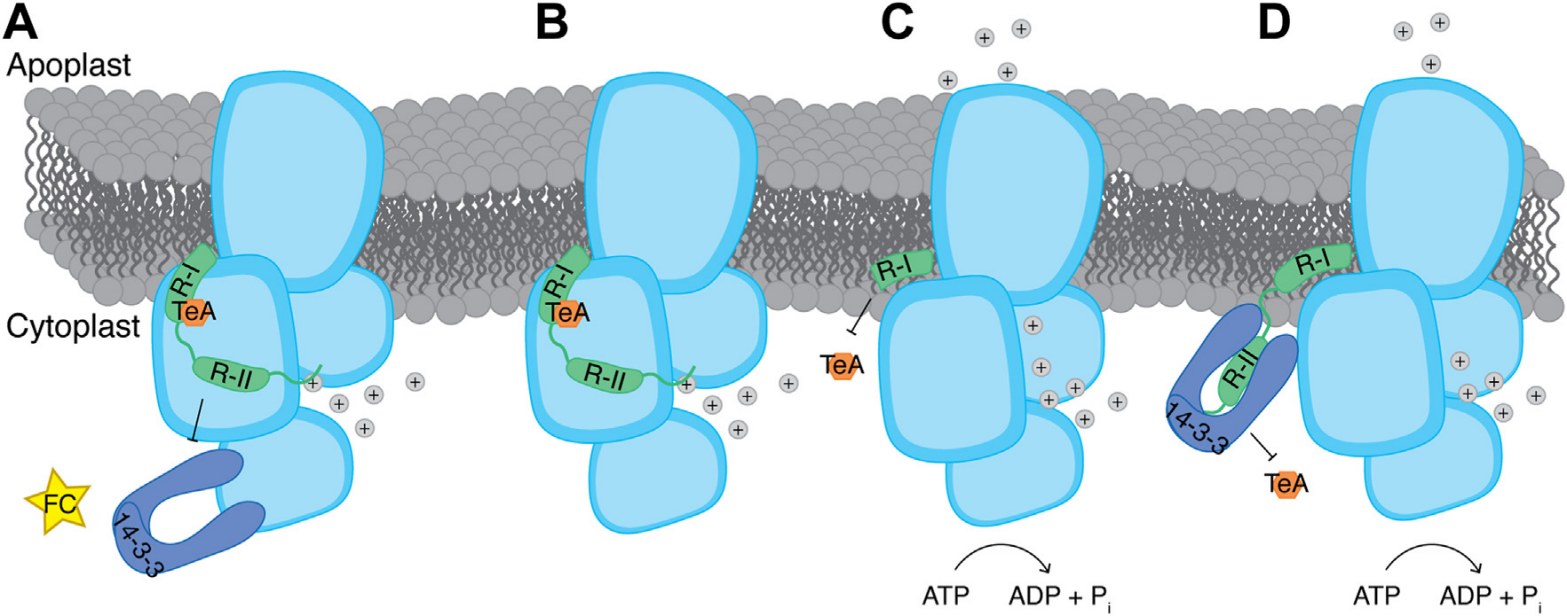
**Model of the role of tenuazonic acid (TeA) in inhibiting plant plasma membrane (PM) H**^**+**^**-ATPase activity.***A*, the binding affinity of TeA to the PM H^+^-ATPase is higher than the binding affinity of fusicoccin (FC). TeA maintains the enzyme in a tight conformation, preventing it from binding to FC to activate the pump. *B*, TeA inhibits the activity of full-length PM H^+^-ATPase by locking the C-terminal regulatory domain, keeping the pump in an autoinhibited state. *C*, truncation of the C-terminal regulatory domain of PM H^+^-ATPase removes its TeA binding site, preventing the pump from being inhibited by TeA. *D*, phosphorylation of the penultimate threonine residue and binding of 14-3-3 protein to the C-terminal domain of PM H^+^-ATPase keeps the enzyme in a conformation where the C-terminal domain is released and flexible. This prevents TeA from binding to PM H^+^-ATPase, resulting in no inhibition.

FC is known to target the PM H^+^-ATPase in plants, and the mechanism is thought to be irreversible (36), thereby stimulating growth for short periods of time. In the current study, treatment with 10 μM FC significantly stimulated root growth and cell elongation over the course of 6 days, and the plants exhibited larger primary leaves than the untreated control plants (Fig. 3, A, B and D). *A. thaliana* seedlings treated with a combination of TeA and FC exhibited the same phenotype as seedlings treated with only TeA. TeA treatment helped overcome FC-induced activation of the PM H^+^-ATPase when *S. oleracea* PMs were treated with a combination of both toxins at the same time in an ATPase assay (Fig. 3*F*). These results suggest that TeA binds to the H^+^-ATPase more rapidly than FC, thereby preventing the combined activation of the PM H^+^-ATPase by FC and 14-3-3 proteins (Fig. 6*A*). Another example of reduced root growth coupled to a reduction in PM H^+^-ATPase activity involves the receptor-like kinase FERONIA and its ligand, the RAPID ALKALIZATION FACTOR (RALF) peptide (29), which significantly reduced root growth *in vivo* (37). TeA has also been reported to inhibit photosystem II (9, 38), which explains the observed reduction in plant growth. A concentration of IC_50_ = 243.4 μM TeA is needed to inhibit photosystem II in *S. oleracea* (38), whereas a concentration of IC_50_ = 0.7 μM TeA is required to inhibit the PM H^+^-ATPase in *S. oleracea* (5). Therefore, the IC_50_ is lower for the plant PM H^+^-ATPase, suggesting that the pump is a more effective TeA target site.

### TeA targets the C-terminal domain of AHA2 in Regulatory Region I

We previously found that the regulatory C-terminal domain plays a central role in the interaction between the plant PM H^+^-ATPase and the phytotoxin TeA, leading to the inhibition of PM H^+^-ATPase. Here we showed that the activity of full-length AHA2 was significantly inhibited by TeA, suggesting that TeA might stabilize the binding between the C-terminal domain and the pump, keeping it in an autoinhibited state (Figs. 4*B* and 6*B*). The PM H^+^-ATPase was not inhibited by TeA when the last 77 amino acid residues of the C-terminal domain were removed (Figs. 4*B* and 6*C*). To further elucidate the binding site of TeA to the plant PM H^+^-ATPase, we employed a range of AHA2 deletion mutants expressed in yeast. TeA effectively and significantly inhibited the plant PM H^+^-ATPase even when up to 66 C-terminal amino acid residues were removed (Fig. 4*B*), suggesting that the target site for TeA on the PM H^+^-ATPase involves part of Regulatory Region I of the C-terminal domain (Figs. 4*A* and 6, *B* and *C*). To further test this hypothesis, we employed the mutant *aha2*R880A, a high-activity-state mutant in which an Arg residue was mutated into an Ala residue. This mutation is positioned within Regulatory Region I of the C-terminal domain of AHA2 (Fig. 4*A*). *aha2*R880A showed higher susceptibility to TeA treatment than AHA2 (Fig. 4*C*). These findings suggest that changing the charged arginine residue to a smaller and more hydrophobic alanine residue allows for more efficient binding of TeA to the C-terminal domain of AHA2. The stabilized binding between the central domain and the C-terminal domain thus contributes to the retention of AHA2 in an inhibited (low activity) state.

### The hyperactivated form of the plasma membrane H^+^-ATPase is less affected by TeA inhibition than the autoinhibited form

In this study, we aimed to elucidate the mechanism of binding between the plant PM H^+^-ATPase and the phytotoxin TeA. We determined that the binding of TeA depends on the autoinhibited state of the pump. We expressed and purified *aha2*R913A, a high-activity mutant version of AHA2, from *S. cerevisiae.* This allowed us to test the influence of post-translational modifications, as we could purify two different AHA2 pools: one containing the non-modified protein (IM fraction) and one containing the AHA2 protein phosphorylated in the regulatory domain (PM fraction). The latter is required for the binding of the regulatory 14-3-3 protein. TeA treatment inhibited WT AHA2 in both the post-translationally activated state and the autoinhibited state (Fig. 5*A*), but interestingly, no inhibition was observed for the *aha2*R913A mutant in the post-translationally activated state (Figs. 5*A* and 6*D*). Knowing that *aha2*R913A is highly phosphorylated compared to WT, we examined the amount of bound 14-3-3 protein in an overlay assay. The *aha2*R913A mutant bound an average of approximately 4-fold more 14-3-3 protein in the overlay assay than WT AHA2 when equal amounts of PM proteins were loaded onto the gel and protein expression of the AHAs was normalized (Fig. 5*C*). These results suggest that the binding of 14-3-3 protein to the C-terminal domain of AHA2, assuring a fully activated pump, decreases TeA-induced inhibition of the PM H^+^-ATPase activity.

The C-terminal domain is thought to bind to the A- and P-domain of the plant PM H^+^-ATPase in the autoinhibited state (23, 24, 39). The 14-3-3 protein will bind upon phosphorylation of the C-terminal domain, and the C-terminal domain will then be reallocated, allowing conformational changes to the protein (20, 21). A recent study by Zhao *et al*. (40) showed that the C-terminal domain of PMA1 in *S. cerevisiae* binds to the P-domain of the H^+^-ATPase and to the lipid bilayer in which the pump is embedded when it is in the autoinhibited conformational state. Although the C-terminal domain of *S. cerevisiae* PM H^+^-ATPases consists of only 38 amino acid residues compared to the 98 amino acid residues of AHA2, the functions of the respective C-terminal domains are likely similar. By activating the PM H^+^-ATPase and releasing the C-terminal domain from the N- and P-domains, TeA can no longer bind to the plant pump, suggesting that a binding site forms between TeA, the C-terminal domain, and the N- or P- domain. We tested this notion by performing a dephosphorylation assay. Indeed, dephosphorylating the PM H^+^-ATPase, which was purified from plant material (*S. oleracea;* Fig. 5*D*) forced the pump into a low-activity state, allowing for the complete TeA-induced inhibition of ATPase activity (Fig. 5*E*).

Altogether, these data support a model in which TeA stabilizes the binding between the C-terminal domain and central domain of the pump (see Fig. 6*B*), thereby maintaining the H^+^-ATPase in its autoinhibited state.

## Experimental procedures

### Chemicals

Tenuazonic acid was purchased from Santa Cruz Biotechnology and solubilized in DMSO. Fusicoccin was purchased from Sigma-Aldrich and solubilized in 97% ethanol.

### Synthesis of tenuazonic acid and L-tenuazonic acid

3-Acetyl-5-(S)-(1-methylpropyl)tetramic acid (tenuazonic acid) was synthesized as described previously (41), except for a slightly different concentration (2.80 mmol) and purification method. The product was purified by reversed-phase preparative HPLC using 0 to 75% MeCN. 3-Acetyl-5-(*S*)-(2-methylpropyl)tetramic acid (vivotoxin II) was synthesized as described previously (41), except for a different concentration (0.79 mmol) and purification method. In addition, the starting material 5-(*S*)-(2-methylpropyl)tetramic acid originated from l-leucine, not l-isoleucine. Part of the product was recrystallized from EtOH and yielded 42 mg of brownish crystals. The rest of the product was purified by reverse-phase preparative HPLC using 0 to 75% MeCN.

### Plant growth

*Herbicide assay: A. thaliana* Col-0 WT seeds were sown on soil pots and incubated for 3 days in the dark at 4 °C. Soil pots with seeds were moved to a growth chamber and incubated for 31 days under short-day conditions (8 h light/16 h dark) at 20 °C. The plants were treated with 1 ml of 200 μM TeA and 0.1% Adigor (adjuvant), 0.1% Adigor, or Milli-Q water every other day for 4 days.

### Sterile plate assays

*A*. *thaliana* Col-0 WT seeds were surface sterilized with chlorine gas in an airtight glass container for 2 h. Seeds were sown on ½× Murashige and Skoog (MS) basal salt mixture (Duchefa) medium (½× MS, 1% sucrose, 0.7% plant agar, pH 5.6 adjusted with KOH) and incubated in the dark at 4 °C for 1 day before being used in the experiments described below.

### Competition studies on root growth

Plates with seeds were moved to a climate chamber under long-day conditions (16 h light/8 h dark) at 20 °C and incubated for 6 days before being transferred to ½× MS medium with TeA and/or FC. The seedlings were incubated for another 6 days before being subjected to bright-field microscopy (Leica DM 5000B); root growth was observed throughout the growth period. Images were used for root measurements in Fiji ImageJ (42).

### Medium acidification assay

Seedlings were incubated for 5 days, transferred to fresh ½× MS medium with 20 μM TeA, and incubated for 3 days. The acidification of the medium was determined using bromocresol purple. Seedlings were transferred to Bromocresol Purple medium (½× MS, 1% sucrose, 0.7% plant agar, and 0.006% bromocresol purple, pH 5.6 adjusted with KOH) after TeA treatment and incubated overnight before assessing acidification.

### Protein expression

AHA2 (pMP1625) and *aha2* mutant proteins *aha2*Δ77, Δ66, Δ61, R880A, R913A (pMP210, 341, 135, 719, 752) were expressed in *S. cerevisiae* RS-72 cells (MATa, *pGal-PMA1*, ade1-100, his4-519, leu2-3, 112) (22, 25, 43, 44). The *S. cerevisiae* RS-72 strain carries the native *PMA1* gene under the control of a galactose-inducible promoter, whereas the expression of *AHA2* was driven by the constitutive *PMA1* promoter. Transformed cells were grown in 5 to 10 ml SGAH medium (20 g/l galactose, 7 g/l yeast nitrogen base without amino acids, 40 mg/l adenine, 20 mg/l histidine and 50 mM succinic acid-Tris, pH 5.5) for 24 h at 30 °C, transferred to 50 to 100 ml fresh SGAH, and incubated for 24 h at 30 °C. The expression of native PMA1 was quenched by transferring the cultures to 1 L YPD (10 g/l yeast extract, 20 g/l Bacto-Tryptone, 20 g/l d(+)-glucose), followed by incubation for 20 h at 30 °C to ensure that AHA2 dominated the cells as the main PM H^+^-ATPase.

### Protein purification

*S. cerevisiae* RS-72 cells were harvested and lysed by vortexing 4× (1 min each) with glass beads (425–600 μm) in homogenization buffer (0.5 M Tris pH 7.5, 5 mM EDTA, 1 mM DTT). Membranes were isolated by ultracentrifugation at 250,000*g* for 1 h at 4 °C and homogenized in STED10 buffer (10% sucrose [w/w], 100 mM Tris pH 7.5, 1 mM EDTA pH 8, and 1 mM DTT). The PM fraction was separated from the total membrane fraction on a sucrose step gradient (10/43/53% STED) by centrifugation at 154,000*g* for 16–20 h at 4 °C. The separated PM and IM were pelleted at 250,000*g* for 1 h at 4 °C, homogenized in GTED20 (20% glycerol [v/v], 100 mM Tris pH 7.5, 1 mM EDTA pH 8, and 1 mM DTT), and flash frozen in liquid nitrogen. PM vesicles were isolated from *S. oleracea* using the two-phase partitioning method as previously described (45). Protein concentrations were determined using Bradford reagent (46) with γ-globulin as the standard reference protein.

### SDS-PAGE and immunoblotting

Proteins were precipitated in 10% TCA and solubilized in 1× SDS loading buffer (62.5 mM Tris-HCl pH 6.8, 2% SDS, 2 mM DTT, 20% STED60, 3 mM bromophenol blue, 2.5 mM EDTA). Membrane proteins were separated by SDS-PAGE following standard techniques, with 10 to 20 μg membrane protein loaded per lane. Proteins were stained with Coomassie Brilliant Blue G-250 (CBB) or blotted onto a nitrocellulose membrane for immunoblot analysis. Immunoblotting was performed using specific primary antibodies for the PM H^+^-ATPase C-terminal domain (1:3000) (47) and pThr residues (1:500) (Agrisera AS18 4171), followed by secondary rabbit IgG alkaline phosphatase-conjugated antibodies (1:5000) (DAKO Denmark A/S), and visualized using BCIP/NBT (Sigma-Aldrich) substrate. Overlay blots were incubated with 2 to 4 μg/ml 14-3-3φ RGS-his-tagged protein in overlay buffer (20 mM Mes, 130 mM NaCl, 10 mM MgSO_4_, 100 μM CaCl_2_, pH 6.5). Primary mouse RGS-his antibody (1:2000) (QIAGEN), and secondary mouse IgG alkaline phosphatase-conjugated antibodies (1:2000) (Sigma-Aldrich) were used to detection of 14-3-3 protein binding, and the reactions were visualized using BCIP/NBT (Sigma-Aldrich) substrate. The relative binding of 14-3-3 protein in the overlay assay was quantified using Fiji ImageJ (42).

### Dephosphorylation of membrane proteins

*S. oleracea* PM proteins were dephosphorylated prior to the ATPase assay, using λ-Protein Phosphatase (New England Biolabs) following the manufacturer’s protocol.

### ATPase activity

The activities of AHA2 and PM H^+^-ATPase mutant proteins expressed in *S. cerevisiae* and *S. oleracea* PM H^+^-ATPase in response to TeA treatment were determined using an ATPase assay as previously described (48) and some modifications (49). The assay was performed using 2.5 mM ATP at pH 6.5 or 7 in Basic ATPase buffer (20 mM MOPS, 8 mM MgSO_4_, 50 mM KNO_3_ [V-ATPase inhibitor], 5 mM NaN_3_ [mitochondrial ATPase inhibitor], and 0.25 mM Na_2_MoO_4_ [acid phosphatase inhibitor]) to achieve the optimum activity of the PM H^+^-ATPase examined in these experiments. Protein levels (μg) used for the assays were quantified using protein expression levels from CBB-stained proteins separated by SDS-PAGE using Fiji ImageJ. This ensured that equal amounts of each AHA2 and mutant protein were used in the individual assays. For assays with *S. oleracea* PM vesicles, 1 μg of protein was used, and vesicles were turned inside out by incubation on ice with 0.05% Brij58 10 min prior to the start of the assays. *S. oleracea* PM vesicles were pre-incubated for 10 min with TeA/FC prior to starting the competition assays. The reactions were incubated for 30 min at 30 °C. The reactions were stopped by the addition of STOP solution (0.15 M ascorbic acid, 0.45 M HCl, 0.9% SDS, 5.5 mM (NH_4_)_6_Mo_7_O_24_) and incubated on ice for 15 min. The reactions were fixed in Na-Arsenate solution (0.15 M NaAsO_2_, 0.08 mM Na_3_C_6_H_5_O_7_, 2% glacial acetic acid). Absorbance was measured at 860 nm (SpectraMax M5) after fixation and an additional 1 h incubation at room temperature.

## Data availability

All data are contained within the article and can be shared upon request.

## Supporting information

This article contains supporting information.

## Conflict of interest

The authors declare that they have no conflict of interest with the contents of this article.

## Figures and Tables

**Supporting Figures 2:**