# Supplementary material for: The mechanism behind tenuazonic acid-mediated inhibition of plant plasma membrane H+-ATPase and plant growth
Source: J Biol Chem. 2024 Mar 13;300(4):107167. doi: 10.1016/j.jbc.2024.107167 (PMC11002603; doi:10.1016/j.jbc.2024.107167)
Supplement: Supporting Information [file mmc1.docx]

**Supporting information**

**Figure S1. CBB-stained SDS-PAGE gels of the plasma membrane (PM) and internal membrane (IM) fractions from *S. cerevisiae*.** AHA2, *aha2*∆77, and *aha2*∆61 (A), AHA2 and *aha2*∆66 (B), AHA2 and *aha2*R880A (C) were expressed in *S. cerevisiae* and purified from the PM and IM fractions. The band just under 100 kDa to 85 kDa represents AHA2 or truncated mutants. Arrows points towards bands used for protein quantification for the ATPase assays.

**Figure S2. Inhibition of the ATPase activity of AHA2 and truncated mutant proteins in treated vs. non-treated membranes.** Tenuazonic acid (TeA)-induced inhibition of ATPase activity by AHA2, *aha2*∆61, *aha2*∆66, and *aha2*∆77 purified from the plasma membrane (PM) fraction from *S. cerevisiae*. Membrane fractions were treated with 50 µM TeA or 50 µM vanadate (P-type ATPase inhibitor) at pH 6.5 for AHA2 and pH 7 for *aha2*∆77, *aha2*∆66, and *aha2*∆61. Assays were performed in technical replicates of three from two biological replicates (*n*=6), and activity is presented as the mean percentage compared to AHA2 non-treated ±SEM.
